# Altered Proteomic Polymorphisms in the Caterpillar Body and Stroma of Natural *Cordyceps sinensis* during Maturation

**DOI:** 10.1371/journal.pone.0109083

**Published:** 2014-10-13

**Authors:** Yun-Zi Dong, Li-Juan Zhang, Zi-Mei Wu, Ling Gao, Yi-Sang Yao, Ning-Zhi Tan, Jian-Yong Wu, Luqun Ni, Jia-Shi Zhu

**Affiliations:** 1 Pharmanex Beijing Clinical Pharmacology Center, Beijing, China; 2 Department of Applied Biology and Chemistry Technology, Hong Kong Polytechnic University, Hung Hom, Kowloon, Hong Kong; 3 Shenzhen TCM Pharmacy and Molecular Pharmacology Kay Laboratory, Hong Kong Polytechnic University, Shenzhen, Guangdong, China; 4 Department of Mechanical and Aerospace Engineering, University of California San Diego, La Jolla, CA, United States of America; 5 NS Center for Anti-Aging Research, Provo, UT, United States of America; Institute for Sustainable Plant Protection, C.N.R., Italy

## Abstract

**Objective:**

To examine the maturational changes in proteomic polymorphisms resulting from differential expression by multiple intrinsic fungi in the caterpillar body and stroma of natural *Cordyceps sinensis* (Cs), an integrated micro-ecosystem.

**Methods:**

The surface-enhanced laser desorption/ionization time-of-flight mass spectrometry (SELDI-TOF MS) biochip technique was used to profile the altered protein compositions in the caterpillar body and stroma of Cs during its maturation. The MS chromatograms were analyzed using density-weighted algorithms to examine the similarities and cluster relationships among the proteomic polymorphisms of the Cs compartments and the mycelial products *Hirsutella sinensis* (Hs) and *Paecilomyces hepiali* (Ph). **Results**: SELDI-TOF MS chromatograms displayed dynamic proteomic polymorphism alterations among samples from the different Cs compartments during maturation. More than 1,900 protein bands were analyzed using density-weighted ZUNIX similarity equations and clustering methods, revealing integral polymorphism similarities of 57.4% between the premature and mature stromata and 42.8% between the premature and mature caterpillar bodies. The across-compartment similarity was low, ranging from 10.0% to 18.4%. Consequently, each Cs compartment (i.e., the stroma and caterpillar body) formed a clustering clade, and the 2 clades formed a Cs cluster. The polymorphic similarities ranged from 0.51% to 1.04% between Hs and the Cs compartments and were 2.8- to 4.8-fold higher (1.92%–4.34%) between Ph and the Cs compartments. The Hs and Ph mycelial samples formed isolated clades outside of the Cs cluster.

**Conclusion:**

Proteomic polymorphisms in the caterpillar body and stroma of Cs change dynamically during maturation. The proteomic polymorphisms in Hs and Ph differ from those in Cs, suggesting the presence of multiple Cs-associated fungi and multiple *Ophiocordyceps sinensis* genotypes with altered differential protein expression in the Cs compartments during maturation. In conjunction with prior mycological and molecular observations, the findings from this proteomic study support the integrated micro-ecosystem hypothesis for natural Cs.

## Introduction

For centuries, *Cordyceps sinensis* has been used as a precious medicinal product in China and other Asian countries and features a broad spectrum of health benefits, including anti-aging and lifespan-extension effects [1]–[3]. (Note: The Latin name *Cordyceps sinensis* (Berk.) Sacc. is used for both the teleomorph/holomorph of *C. sinensis* fungus and the wild product indiscriminately [4],[5]. The fungus was re-named *Ophiocordyceps sinensis* (Berk.) Sung et al. [6]; however, the Latin name for the wild product has remained unchanged. Because a consensus Latin name for the wild product has not been reached by mycological and TCM botanical taxonomists, we have temporarily used the term *O. sinensis* to refer to the fungus/fungi and continued to use the name *C. sinensis* to refer to the wild product.) Mycological and molecular approaches have demonstrated that *C. sinensis* comprises more than 90 intrinsic fungi from more than 37 genera and at least 6 *O. sinensis* genotypes [7]–[25]. Although an anamorph-teleomorph connection between *Hirsutella sinensis* and *O. sinensis* has been proposed based on the aggregation of indirect evidence [4]–[5],[24], integrated analyses have demonstrated large dissimilarities between the random amplified polymorphic DNA (RAPD) polymorphisms of *H. sinensis* and of the *C. sinensis* ascocarp and no studies to date have truly satisfied Koch's postulates by describing the successful artificial induction of *C. sinensis* sexual fruiting bodies and ascospores [9],[17]–[18],[25]–[29]. However, there has been no direct evidence to either approve or reject the *Paecilomyces hepiali* hypothesis for the *O. sinensis* anamorph [30]. *P. hepiali, H. sinensis* and several mutant genotypes of *O. sinensis* have been found to naturally coexist in the ascocarps and ascospores of natural *C. sinensis*, and the fungal complex showed a 39-fold enhancement of its infection potency over that of pure *H. sinensis* [31]–[32]. Other researchers have thus hypothesized that *C. sinensis* is an integrated micro-ecosystem with differential expressions by multiple intrinsic fungi in its compartments and have identified a culture-dependent microbial community or mycobiota in natural *C. sinensis* along with evidence of possible symbiotic interactions among the component fungi [13]–[23],[27]–[32]. We have previously reported dynamic changes in the differential fungal expression of at least 6 *O. sinensis* genotypes during *C. sinensis* maturation [19]–[23],[29]. However, no previous studies have compared the proteomes of *C. sinensis* and *H. sinensis* (the proposed anamorphic fungus of *O. sinensis*) or reported global changes in the macrocosmic proteomic polymorphisms in *C. sinensis* compartments during maturation. In contrast to the microcosmic studies that have focused specifically on individual protein species, we used the surface-enhanced laser desorption/ionization time-of-flight mass spectrometry (SELDI-TOF MS) protein chip technique in this study to macrocosmically profile the changes in proteomic polymorphisms in the *C. sinensis* caterpillar body and stroma during maturation [33]–[34]. We also examined the similarities and cluster relationships between the proteomic polymorphisms of *C. sinensis* and those of the mycelial fermentation products *H. sinensis* Bailing and *P. hepiali* Cs-4.

## Materials and Methods

### Collection of *C. sinensis*

**Figure 1:**
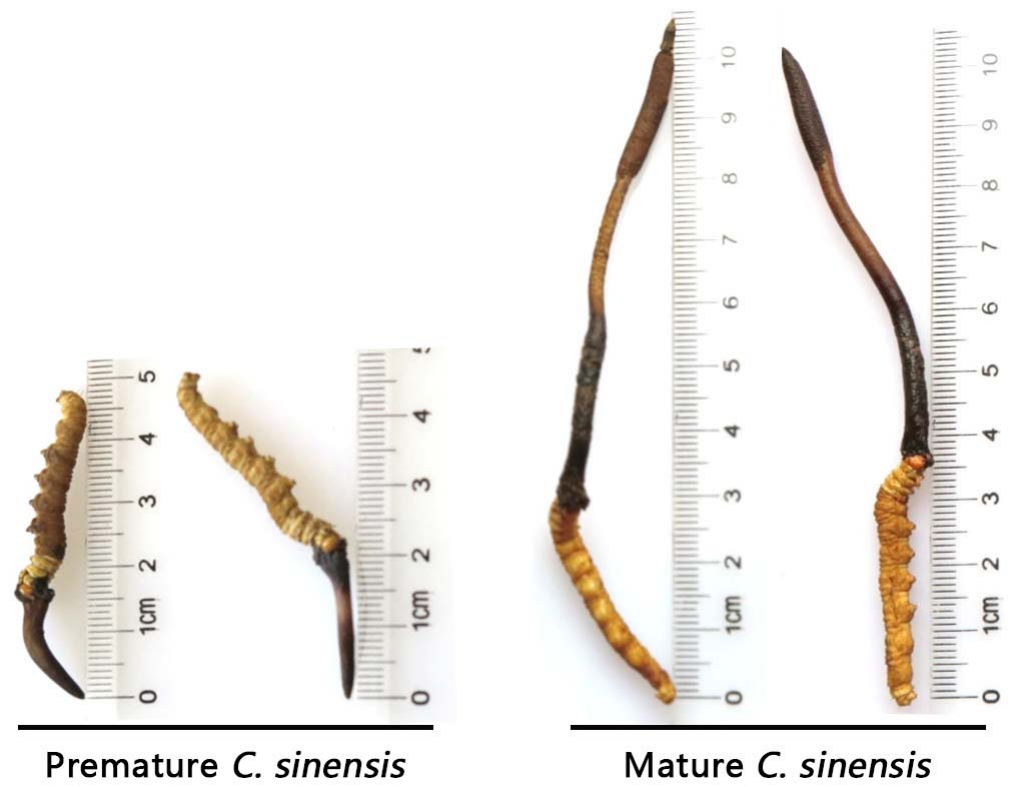
Freshly collected natural *C. sinensis* at 2 maturation stages with stromata of various heights.

Fresh *C. sinensis* specimens were purchased in a local market (Latitude 30°04′N, Longitude 101°95′E) in the Kangding County of Sichuan Province, China. Governmental permission was not required for *C. sinensis* purchases in local markets, and the collections of *C. sinensis* specimen sales by local farmers fall under the governmental regulations for traditional Chinese herbal products. Premature *C. sinensis* features a plump caterpillar body (sclerotium) and a short stroma ranging from 1.0 to 2.0 cm in length (Figure 1). Mature *C. sinensis* features a less plump caterpillar body and a long stroma with a length of>5.0 cm and an expanded portion densely covered with ascocarps close to the stroma tip. All fresh *C. sinensis* specimens were washed thoroughly on site in running water with gentle brushing, soaked in 0.1% mercuric chloride for 10 min for surface sterilization and washed again 3 times with sterile water. The specimens were immediately frozen in liquid nitrogen for transportation and storage prior to further processing in the lab in Beijing [13].

### Sample preparations for proteomic profiling

Ten *C. sinensis* specimens at each maturation stage were used in this study. The caterpillar bodies and stromata from the premature and mature *C. sinensis* specimens and the mycelial fermentation products *H. sinensis* Bailing (Bailing capsule, Lot #040811, #050403 and #051103, Zhejiang American-Sina Pharmaceutical Company, Hangzhou, Zhejiang, China) and *P. hepiali* Cs-4 (Jinshuibao capsule, Lot #JX12931, #20040608 and #20051020, Jiangxi TCM Pharmaceutical Company, Nanchang, Jiangxi, China) were individually ground into powder in liquid nitrogen. To evaluate the proteomic polymorphisms of the samples as group-averages at each maturation stage to minimize the influence of individual variations due to sampling and the lack of a more accurate method to measure the sample's maturation status, the powders (0.5 g each) of the *C. sinensis* compartment samples were pooled according to their compartment origins and maturational stages to form the following testing samples: premature stroma, premature caterpillar body, mature stroma and mature caterpillar body. Based on the pre-test results with insignificant variations in overall proteomic similarity, 0.95 for the 3 *H. sinensis* Bailing samples and 0.96 for the 3 *P. hepiali* Cs-4 samples, the powders of *H. sinensis* Bailing (Lot #051103) and *P. hepiali* Cs-4 (Lot #20051020) were selected for the formal study. The powder samples were dissolved in 600 µl of tris-glycine buffer (pH 8.3) and centrifuged at 14,000 rpm for 5 min at 4°C. The sample supernatants were used for proteomic profiling.

### SELDI-TOF mass spectrometry

The supernatants prepared above were diluted in PBS to a concentration of 200–300 nM before application to a normal-phase biochip and analysis on a PBS-II protein chip reader (SELDI-TOF MS; BioSpace Ciphergen Biosystems, Fremont, CA, USA) [33]–[34]. The SELDI-TOF MS experiments were performed at the Universities' Confederated Institute for Proteomics at the School of Life Sciences, Beijing Normal University, Beijing, China. In brief, different proteins captured on the surface of protein chips were collected through SELDI-TOP mass spectrometry using a laser power of 215 (sensitivity 9; molecule size range: 0–60,000 Da). Following mass calibration, total ion current normalization and baseline subtraction, the molecular size ranges of proteins were manually selected for analyses, and the intensities (peak heights) were extracted using ProteinChip software (Ciphergen proteinchip 3.0.2).

### Across-chromatogram normalization of densities of protein species

**Figure 2:**
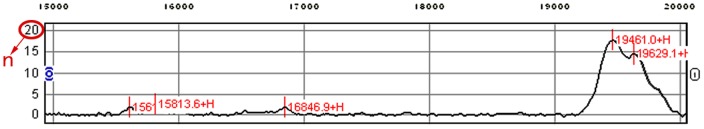
A representative SELDI-TOF MS chromatogram tracing. The tracing scale maximum, n, on the vertical axis of each chromatographic panel was used as the across-chromatogram normalization reference.

The SELDI-TOF MS chromatograms were scanned with Quantity One software (Bio-Rad Laboratories, Inc., Hercules, CA, USA). To conduct integrated proteomic profiling on the basis of chromatographic tracing at the molecular weight segments, the band trace quantities (OD*mm) of all protein bands in all chromatograms were normalized using the maximal chromatographic tracing scales for each molecular weight segmented tracing panel as the reference factor (Figure 2). The relative intensity/density was defined as the scanned band trace quantity (OD*mm) multiplied by the difference between the maximum scale “n” on the vertical axis of each chromatographic tracing panel and the baseline scale if the trace baseline was not exactly zero.

### Similarity computation for proteomic polymorphisms

ZUNIX equations (http://www.ebioland.com/ZUNIX.htm; Beijing Bioland Technology, 2013) were used for similarity computations with band intensities/density weighting [28]–[29] while considering (i) mismatched protein bands and (ii) matched protein bands with dissimilar intensities/densities. The following ZUNIX equation (1) was used to compare the polymorphisms of 2 mass spectrometry chromatograms: *d_ik_*≥0, *i* = 1,2, *k* = 1,2, …, m. We defined the measure of similarity as follows:
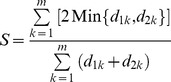
(1)where the similarity of the 2 densities *d*_1*k*_ and *d*_2*k*_ is defined as the common portion of their values.

The second ZUNIX equation (2) is suitable for comparing the proteomic polymorphisms in more mass spectrometry chromatograms, where *d_ik_≥0*, *i = 1,2*,…,n, *k = 1,2*,…,m and the description is as follows:
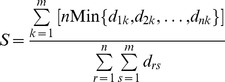
(2)


### Density-weighted cluster analysis for the polymorphic protein fingerprints

For the mismatched protein species, a missing band at the given molecular weight location in a MS chromatogram was assigned a score of 0. The digital density data of all matched and unmatched protein bands in the compared chromatograms were ranked and arbitrarily assigned scores of 1–9 according to the ranks of their densities in 2 or more compared chromatograms [28]–[29]. The digital data scores were entered into PAUP 4.0B (Swofford, 2002; Sinauer Asso. Inc, Sunderland, MA, USA) to construct cluster trees (semi-quantitative density-weighted neighbor-joining distance method; bootstrap  = 1000). In addition to the semi-quantitative algorithm provided by PAUP 4.0B, a fully quantitative cluster analysis was also performed with a parametric hierarchical clustering analysis (density-weighted furthest neighbor Pearson correlation average linkage distance method) in SPSS 10.1 (SPSS Inc., Chicago, IL, USA; Note: no bootstrap strategy was provided in the software package).

## Results

### Comparison of the protein fingerprint chromatograms of premature and mature *C. sinensis*

**Figure 3:**
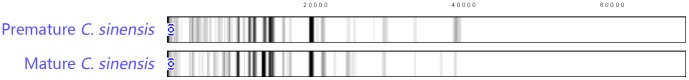
SELDI-TOF MS protein chromatograms to examine the protein fingerprints (molecular weight: 0 to>60,000 Daltons) and proteomic polymorphisms of premature and mature *C. sinensis*.

Figure 3 displays the SELDI-TOF MS chromatograms for the *C. sinensis* protein species at the 2 maturation stages in a molecular weight range of 0 to>60,000 Daltons. Using the density-weighted ZUNIX equation (1), a percentage similarity of 57.9% was observed between the protein fingerprint polymorphisms of pooled premature and mature *C. sinensis* samples, thus indicating altered protein expression during *C. sinensis* maturation.

### Comparison of the polymorphic protein chromatograms of the caterpillar bodies and stromata of premature and mature *C. sinensis*

**Figure 4:**
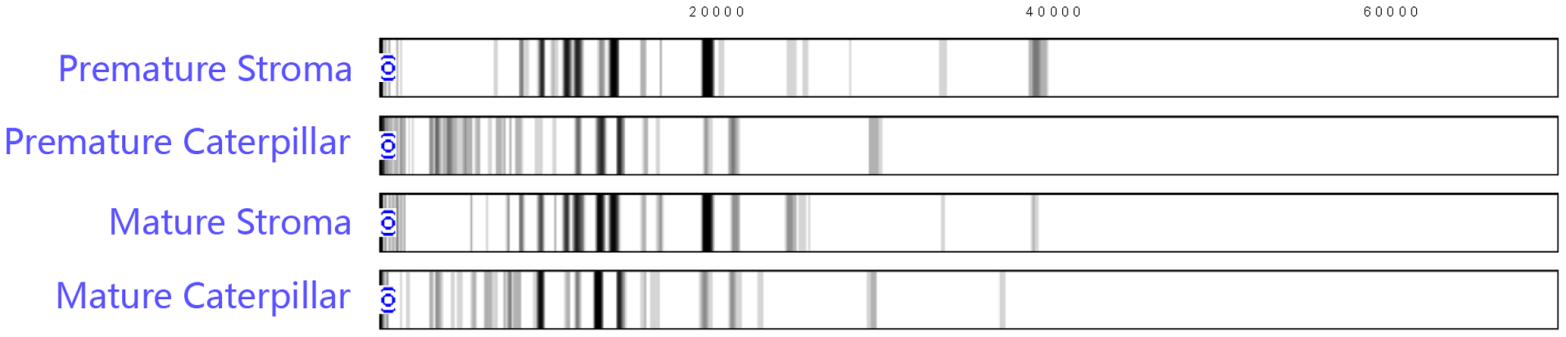
SELDI-TOF MS protein chromatograms to examine the protein fingerprints (molecular weight: 0 to>60,000 Daltons) and proteomic polymorphisms of the caterpillar bodies and stromata of premature and mature *C. sinensis*.

Figure 4 displays the SELDI-TOF MS chromatograms for the *C. sinensis* caterpillar body and stroma protein moieties at the 2 maturational stages in a molecular weight range of 0 to>60,000 Daltons. Using ZUNIX equation (2), an overall percentage similarity of 3.1% was observed between the proteomic polymorphisms for all *C. sinensis* caterpillar body and stroma samples at both maturation stages. Using ZUNIX equation (1) for pairwise comparisons, the calculated similarities from Figure 4 were 57.4% and 42.8% between the proteomic polymorphisms of the 2 maturation stages in the stromata or caterpillar bodies, respectively, but were much lower (10.0%–18.4%) for the across-compartment pair comparisons (Table 1). These similarities indicate major differences in the proteomic profile within the *C. sinensis* caterpillar body and stroma resulting from large differences in the compositions of the multiple intrinsic fungi from more than 37 genera and at least 6 mutant *O. sinensis* genotypes together with the transcription and translation of their fungal genes. In contrast to the large between-compartment differences in the proteomic profile, the within-compartment differences were moderate across the *C. sinensis* maturation stages.

**Table 1:** Percentage similarities in the total protein profiles of the caterpillar bodies and stromata of the premature and mature *C. sinensis*, computed with the density-weighted ZUNIX equation (1).

	Stroma		Caterpillar Body	
	Premature	Mature	Premature	Mature
Premature stroma	—	—	—	—
Mature stroma	57.4%	—	—	—
Premature caterpillar body	10.0%	13.2%	—	—
Mature caterpillar body	18.4%	17.8%	42.8%	—

### Comparison of the *C. sinensis*, *H. sinensis* and *P. hepiali* sample protein fingerprint chromatograms in multiple molecular weight segments

**Figure 5:**
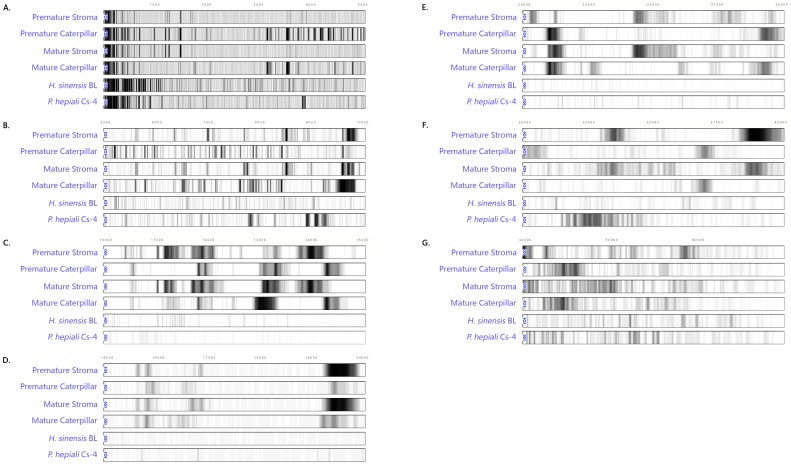
SELDI-TOF MS protein chromatograms to examine protein polymorphisms in the stroma and the caterpillar body specimens of premature and mature *C. sinensis* and the mycelial fermentation products *H. sinensis* Bailing and *P. hepiali* Cs-4. Total proteins were extracted from the caterpillar bodies or stromata of natural *C. sinensis* at 2 maturation stages and from the mycelial products *H. sinensis* Bailing (BL) or *P. hepiali* Cs-4. Panels A, B, C, D, E, F and G present proteins with molecular weights ranging from 0–5,000, 5,000–10,000, 10,000–15,000, 15,000–20,000, 20,000–30,000, 30,000–40,000 and 40,000 to>60,000 Daltons, respectively.

The above-described results for the *C. sinensis* proteins are displayed integrally from molecular weights of 0 to>60,000 Daltons, as shown above in Figures 3 and 4. To increase the chromatographic resolution, Figure 5 displays 7 panels of the segmented SELDI-TOF MS chromatograms of all protein species in the *C. sinensis* caterpillar bodies and stromata at the 2 maturation stages as well as of the commercial mycelial fermentation products *H. sinensis* Bailing and *P. hepiali* Cs-4. Figure 5-A presents protein species in the molecular weight range from 0 to 5,000 Daltons, Figure 5-B ranges from 5,000 to 10,000 Daltons, Figure 5-C ranges from 10,000 to 15,000 Daltons, Figure 5-D ranges from 15,000 to 20,000 Daltons, Figure 5-E ranges from 20,000 to 30,000 Daltons, Figure 5-F ranges from 30,000 to 40,000 Daltons and Figure 5-G ranges from 40,000 to>60,000 Daltons.

The segmented mass spectrometry chromatograms shown in Figure 5 indicate large polymorphic differences between the protein profiles of the *C. sinensis* compartments at each of the 2 maturation stages, and the complex protein expression patterns resulting from multiple intrinsic fungi across the *C. sinensis* compartments underwent differential maturational fungal expression changes. The mass spectrometry chromatograms also indicate large overall differences between the proteomic polymorphisms of *C. sinensis* and those of the fermentation products *H. sinensis* Bailing and *P. hepiali* Cs-4.

### Polymorphic similarities in the protein fingerprints of the *C. sinensis* compartments at 2 maturational stages and the mycelial fermentation products *H. sinensis* Bailing and *P. hepiali* Cs-4

The densities of all protein species in all 7 mass spectrometry chromatogram panels shown in Figure 5 were normalized using the mass spectrometry tracing scales described in the Methods section and illustrated in Figure 2. The normalized densities were subjected to polymorphic similarity calculations with ZUNIX similarity equation (1) [28]–[29], to examine the similarities between the protein profiles of the *C. sinensis* compartment and those of the mycelial products *H. sinensis* Bailing and *P. hepiali* Cs-4. As shown in Table 2, the proteomic polymorphism similarities were low (0.51%–1.04%) between the *H. sinensis* Bailing and *C. sinensis* protein profiles and were 2.8- to 4.8-fold higher (1.92%–4.34%) between the *P. hepiali* Cs-4 and *C. sinensis* compartments than between the *H. sinensis* Bailing and *C. sinensis* compartments.

**Table 2:** Percentage similarities in proteomic polymorphisms between the *C. sinensis* compartment samples at 2 maturational stages and *H. sinensis* Bailing (BL) and *P. hepiali* Cs-4.

		Stroma		Caterpillar body		
		Premature	Mature	Premature	Mature	*H. sinensis* BL
Percentage similarity (%)	*H. sinensis* BL	0.69%	0.51%	1.04%	0.87%	—
	*P. hepiali* Cs-4	1.92%	2.33%	4.34%	4.18%	6.52%
Similarity Ratio (fold)	(*P. hepiali* Cs-4 *vs. H. sinensis* BL)	2.8-fold	4.5-fold	4.2-fold	4.8-fold	

### Density distributions of all protein bands and determination of the scoring cutoff value for the semi-quantitative analysis

**Figure 6:**
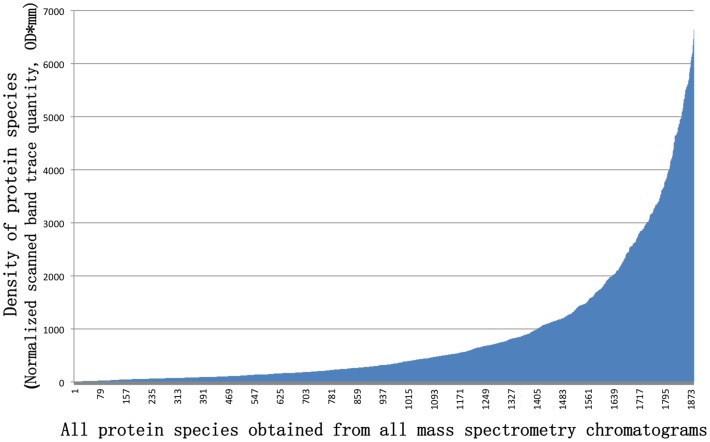
Distribution of the normalized scanned band trace quantities (OD*mm) of approximately 1,900 protein species from all segmented SELDI-TOF MS protein chromatograms in **Figure 5**.

After normalization, the scanned band trace quantities (OD*mm) of approximately 1,900 protein bands were sorted for arbitrary scoring in preparation for the cluster construction using the semi-quantitative density-weighted algorithm (Figure 6).

### Density-weighted cluster analysis of the protein fingerprints of *C. sinensis* and the mycelial products *H. sinensis* Bailing and *P. hepiali* Cs-4

**Figure 7:**
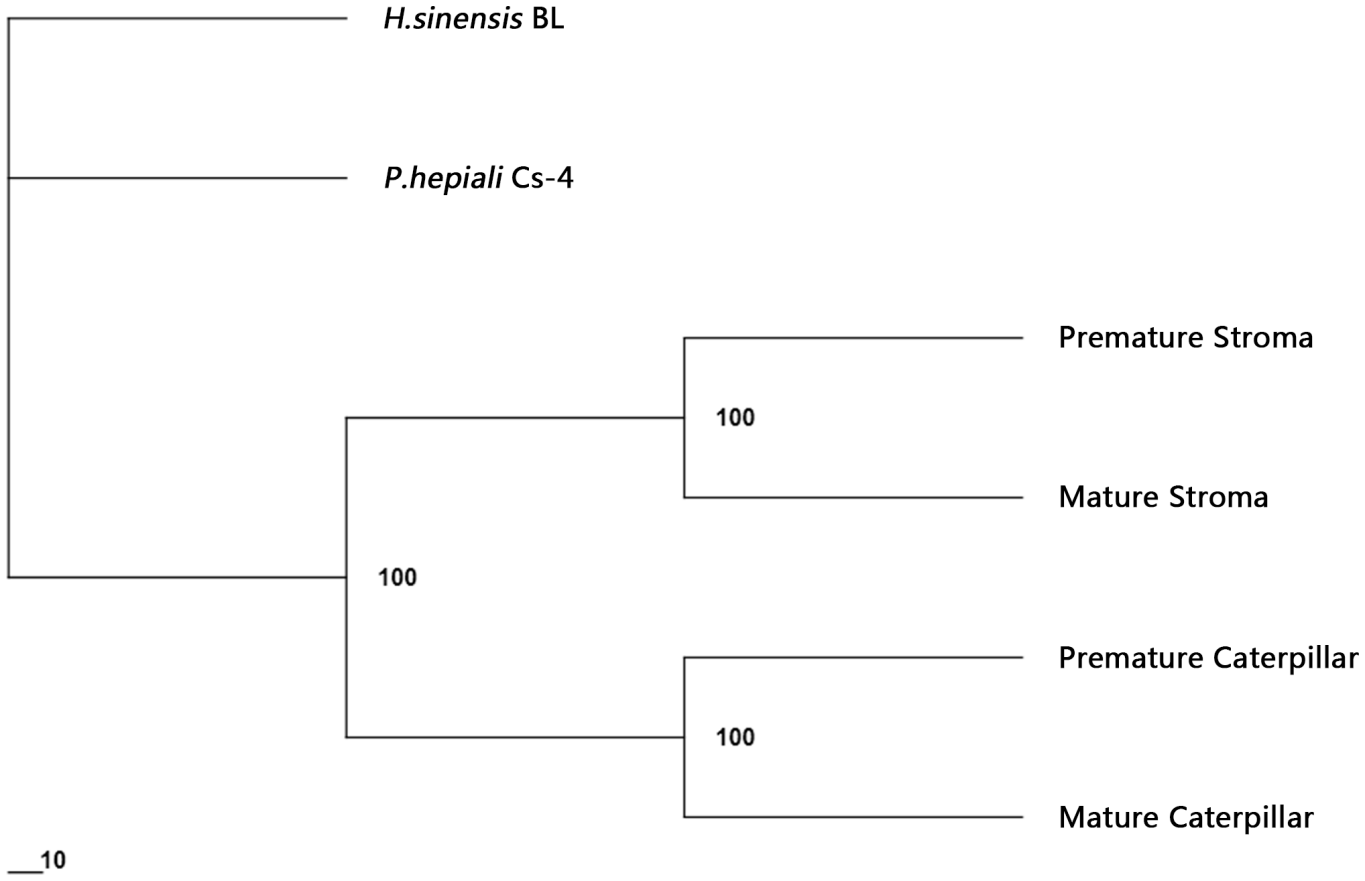
Integral cluster tree of all the proteomic chromatograms constructed with the semi-quantitative density-weighted algorithm. “*H.sinensis* BL” refers to the *H. sinensis* Bailing mycelial product, “*P.hepiali* Cs-4” refers to the *P. hepiali* Cs-4 mycelial product, and “Caterpillar” refers to the caterpillar body. Each protein species from all proteomic chromatograms in Figure 5 was assigned a score of 1–9 based on its density rank among the densities of all compared protein species; the missing protein band at the same molecular weight was assigned a score of 0. All protein species from the chromatograms in Figure 5 were entered into the cluster construction using the neighbor-joining distance method (bootstrap  = 1000).

The highest density value in Figure 6 was divided by 9 to obtain the critical cut-off values for a semi-quantitative density grouping. Each density was assigned a score from 1 to 9 according to the above-mentioned cutoff values, and all arbitrarily assigned scores were used for the cluster construction according to the density-weighted algorithm provided by PAUP 4.0B software [28]–[29]. Figure 7 displays a cluster tree that was constructed with the density-weighted neighbor-joining algorithm (bootstrap  = 1000). Similar to the percentage similarity results shown in Table 1, the caterpillar body samples formed 1 clade and the stroma samples formed another clade; these 2 clades then formed a *C. sinensis* cluster. The mycelial fermentation product *H. sinensis* Bailing formed an isolated clade that was separated from the *C. sinensis* cluster by the clade formed by *P. hepiali* Cs-4.

**Figure 8:**
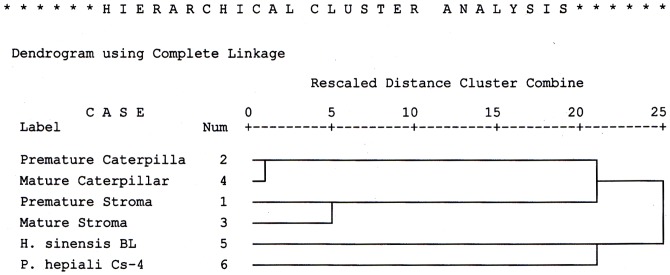
Integral cluster tree of all proteomic chromatograms constructed with the fully quantitative density-weighted algorithm. “H. sinensis BL” refers to the *H. sinensis* Bailing mycelial product; “Caterpillar” or “Caterpillar” refers to the caterpillar body. All protein species from all chromatograms in Figure 5 were entered into the cluster construction, using the furthest neighbor (Pearson correlation average linkage) method of hierarchical cluster analysis.

Although the PAUP 4.0B software offered the advantage of constructing cluster trees according to the bootstrap value (bootstrap  = 1000), the program used only semi-quantitative algorithms. A fully quantitative, density-weighted algorithm included in the SPSS 10.1 software package was also employed to construct a cluster tree [28]. As shown in Figure 8, the *C. sinensis* sample clade formation pattern for *C. sinensis* samples generated using the fully quantitative algorithm was similar to that generated via semi-quantitation, as shown in Figure 7. The caterpillar body and stroma clades joined to form a *C. sinensis* cluster with a greater rescaled distance in the cluster tree that was indicative of large differences in polymorphic protein expression between the *C. sinensis* caterpillar bodies and stromata and reflective of the low similarity observed between the *C. sinensis* compartments in Table 1. The mycelial fermentation products *H. sinensis* Bailing and *P. hepiali* Cs-4 formed a clade with a greater rescaled distance and were thus situated outside of the *C. sinensis* cluster.

## Discussion

*C. sinensis* is one of the most valued Chinese medicinal products. This organism grows only in areas of high elevation on the Qinghai-Tibetan Plateau and features a complex life cycle. Studies have reported that *C. sinensis* comprises more than 90 intrinsic fungal species from more than 37 genera and at least 6 genotypes of *O. sinensis* fungi [7]–[23]. Of these, the most abundant culturable fungi are *Pseudogymnoascus roseus* in the sclerotia and cortices and *Penicillium chrysogenum* in the stromata, as reported by Zhang et al. [17]. Previously, we reported that *C. sinensis* maturation was associated with dynamic changes in the intrinsic fungal species and mutant *O. sinensis* genotypes along with significant changes in the RAPD molecular marker polymorphisms and component chemicals [13],[19]–[23],[29]. The fungal background of *C. sinensis* becomes even more complex when non-culturable fungal species are considered [18]–[23]. These findings reflect the altered fungal expression of multiple intrinsic fungi and support the hypothesis that *C. sinensis* is an integrated micro-ecosystem of multiple intrinsic fungi, as proposed by Liang *et al*. [27]. Density-weighted algorithms for similarity computations and cluster constructions were used in this study to analyze the mass spectrometry chromatograms of polymorphic proteomes, the downstream transcription/translation products of multiple fungal genomes. We observed different proteomic profiles with similarities of 10.0% between the premature caterpillar bodies and stromata and 17.8% between the mature caterpillar bodies and stromata of *C. sinensis* (*cf.* Figure 4; Table 1), consistent with the mycological and molecular observations of diverse fungal populations in the two *C. sinensis* compartments [17]–[18]. However, considerably great proteomic polymorphism similarities of 42.8% and 57.4% were observed within the *C. sinensis* caterpillar body and stroma, respectively, at the 2 *C. sinensis* maturation stages (*cf.* Figure 4; Table 1). The differences in the across and within-compartment similarities between the proteomic profiles might possibly be derived from 2 major factors: (1) differential protein expression of the multiple fungal genomes (multiple mutant *O. sinensis* genotypes and the multiple intrinsic mesophilic and psychrophilic fungi), of which part or all undergo maturational alterations, and (2) protein species from the dead bodies of the *C. sinensis* ghost moth larvae, which are not merely a group of nutrients for fungal growth but also as a part of the species complex, along with all of the previously reported small chemical components [13],[38]–[47], contribute to the overall pharmacology of the natural medicinal product and partly explain the various therapeutic potencies of premature and mature *C. sinensis* that have been identified by traditional Chinese medicine quality grading system.

Density-weighted algorithms for similarity computations and cluster constructions were used to compare RAPD molecular marker polymorphisms in previous studies of *C. sinensis* [28]–[29]. Although density-unweighted arithmetic methods have been widely used in literature, these methods are only suitable for the analyses of “all-or-none” data. The density-weighted arithmetic methods used in this proteomic study are more mathematically general and sufficiently sensitive to capture all of the detailed information regarding dynamic changes in proteins expressed by the various intrinsic fungi during *C. sinensis* maturation [28]–[29]. These algorithms provide scientists with accurate analytical means with which to trace changes in the proteomic and molecular marker polymorphisms in natural *C. sinensis*.

In this first study testing the proteomic polymorphisms of different compartments of wild *C. sinensis* during maturation, the overall proteomic polymorphisms were compared in the pooled samples of 10 *C. sinensis* specimens of each maturation stage. The height of the *C. sinensis* stroma (*cf*. Figure 1) has been taken as the standard for the potency-quality grading of natural *C. sinensis* on the market. Such a common practice for potency grading can be explained by a *C. sinensis* mycology expert that the premature *C. sinensis* with a short stroma grows asexually, whereas the long-stroma *C. sinensis* with the formation of the ascocarp portion (the expanded portion close to the tip of the stroma) primarily grows sexually (personal communication with Prof. YL Guo). According to the comments of Prof. Guo regarding the asexual-sexual growth of *C. sinensis*, our previous molecular systematic studies demonstrated that the maturation of wild *C. sinensis* is a continuous biological course along with the weather during spring. However, there is no existing accurate method thus far to measure sample's maturation status. We also found previously large differences in the fungal activity of *H. sinensis*, biomasses of the fungus-specific DNA species, and small organic chemicals in wild *C. sinensis* during maturation, indicating large differences in fungal expression during *C. sinensis* maturation [13],[19]–[23],[28]–[29]. Therefore, we designed this study with 2 special sample arrangements of the test materials to minimize variations in individual specimens: (1) the selection of *C. sinensis* specimens with the clear morphological characters shown in Figure 1, i.e., very premature *C. sinensis* with a short possible stroma (1-2 cm) and mature *C. sinensis* with a long stroma (>5 cm) and with definite formation of the ascocarp portion; and (2) assessment of the pooled samples (10 specimens at each maturation stage; stromata and caterpillar bodies separated from the same specimens). In addition to the examination of maturational and compartmental group differences in proteomic polymorphism within a *C. sinensis* population, it is possible that there are individual differences in some degree in proteomic polymorphism within a *C. sinensis* population and within maturation groups. These individual differences are likely due to differences such as in the instar and nutrition status of the larvae of ghost moths within the family Hepialidae at the time of fungal infection, the growth location and environment (e.g., elevation and temperature, strength of plateau wind and sunshine in the growth area, amount of snow in winter and rain in spring, soil fertility, surrounding vegetation), the total weight and length of the *C. sinensis* specimens, and the weight ratio and height ratio of the caterpillar body and stroma of individual *C. sinensis* specimens. This study design of pooling samples, however, is limited regarding the exploration of such individual variations at each estimated maturation stage. However, there may also be population differences among the *C. sinensis* specimens collected from different production areas, likely due to the different species of larvae of ghost moths within the family Hepialidae, differences in latitude, possibly different local soil fungal flora or mycobiota and other environment factors. All these considerations should encourage future studies to further explore variations in the molecular and proteomic polymorphisms and chemical profiles among the individual *C. sinensis* specimens collected within a production area or among the *C. sinensis* populations from various production areas. Perhaps prior to the future comparison of individual specimens, an accurate method for determining *C. sinensis* maturation stages may need to be established with the combined use of morphological characters and molecular markers to distinguish proteomic variations due to slightly different maturation stages of *C. sinensis* specimens or due to true differences in protein expression in individual specimens at the same maturation stage. To this end, fungal biomass ratios, for example, GC-biases *vs*. AT-biases of *O. sinensis*, may serve as a molecular marker to assist the morphological characterization when determining the *C. sinensis* maturation status [15]–[16],[19]–[23].

*H. sinensis* has been proposed as an anamorph of *O. sinensis*; natural *C. sinensis* is considered a single fungus product [4]–[5]. These hypotheses were proposed based on the aggregation of indirect evidence, such as morphological findings for the isolates from natural *C. sinensis*, ITS sequencing and results from microcycle conidiation of ascospores under particular culture conditions [4]–[5],[24]. No scientific studies to date have truly satisfied Koch's postulates, which have demonstrated the successful artificial induction of the *C. sinensis* sexual fruiting body and ascospores [5],[9],[24]–[27],[35]. Shen *et al*. [36] reported extremely slow growth (approximately 2 cm after 7 months) of artificial *Cephalosporium dongchongxiacae* (≡ *H. sinensis*; [4],[36]) fruiting bodies and observed regular, fine and deep twills on the surfaces of long, conically shaped fruiting bodies. The overall appearance of the artificial fruiting bodies, unfortunately, was distinct from that of natural *C. sinensis*, which has a long, round and cylindrical stroma with vertical fine wrinkles, as described in the Chinese Pharmacopoeia. Shen *et al*. [36] also reported the production of ascospores from one of the artificial *C. dongchongxiacae* fruiting bodies that featured no morphological formation of a *C. sinensis*-like ascocarp, the sexual organ of *C. sinensis*, thus indicating an overall teleomorphic morphology distinct from that of natural *C. sinensis*. Shen *et al*. [36] characterized in their paper the unique teleomorphic features of *C. dongchongxiacae*, and the results actually negated the anamorph-teleomorph connection between *C. dongchongxiacae* (≡ *H. sinensis*) and *O. sinensis* in accordance with Koch's postulates. In addition to the dramatic dissimilarities in the RAPD molecular marker polymorphisms between the *C. sinensis* ascocarp and *H. sinensis*, the drastically different proteomic polymorphisms in *C. sinensis* and *H. sinensis* might not support the “single-fungus” hypothesis of *C. sinensis* or the hypothesis of an anamorph-teleomorph connection between *H. sinensis* and *O. sinensis* [13],[25],[28]–[29],[37] (*cf.* Figure 5 and Table 2). Based on these microcosmic and macrocosmic studies, Liang *et al.* [27] hypothesized that *C. sinensis* is an integrated micro-ecosystem with varying compositions of multiple intrinsic fungi. In fact, the coexistence of these multiple fungi has been demonstrated in the culture-dependent and independent microbial communities or mycobiota present in natural *C. sinensis*, along with evidence of symbiotic interactions among the component fungi; these likely represent the key biological actions essential to the natural or artificial production of sexual fruiting bodies and ascospores [17]–[18],[31]–[32].

Studies of *C. sinensis* have detected several groups of chemical components, including carbohydrates; galactomannan; nucleosides; proteins, polypeptides, oligopeptides, polyamines, and diketopiperazines (cyclo-dipeptides); non-hormone sterols; fatty acids and other organic acids; and vitamins and inorganic elements [1],[38]–[40]. Other compounds such as verticiol, acid deoxyribonuclease, myriocin, 3-deoxyadenosine (coydycepin) and cordysinins A–E have also been detected [41]–[45]. Chemical constituent fingerprinting techniques have been used together with similarity comparisons and cluster constructions in *C. sinensis* studies to demonstrate the high similarity of *C. sinensis* samples collected from different production areas [13],[38],[40]–[47]. A cluster analysis of these small organic chemicals via capillary electrophoresis technology demonstrated that several mycelial fermentation products were situated in different clades outside of the cluster containing the natural *C. sinensis* samples collected from different production areas on Qinghai-Tibetan Plateau [40]. However, when analyzing the small chemicals of several natural *C. sinensis* samples collected from Tibet or Qinghai Provinces and of fermentation products (*P. hepiali* Cs-4 and *H. sinensis* Bailing) through HPLC fingerprinting, the clades of natural *C. sinensis* were much closer in rescaled distance to the clade containing the *P. hepiali* Cs-4 products than to the clade containing the *H. sinensis* Bailing products [38]. In the proteomic fingerprint analysis conducted in the current study, in which the bootstrap strategy (bootstrap  = 1,000) was used in the density-weighting algorithm, the fermented products *P. hepiali* Cs-4 and *H. sinensis* Bailing were situated in an isolated clade outside of the *C. sinensis* cluster with the possibility that *P. hepiali* Cs-4 was closer to the *C. sinensis* cluster than was *H. sinensis* Bailing, as shown in Figure 7. This possibility was supported by the 2.8- to 4.8-fold higher similarities of the proteomic polymorphisms between the *P. hepiali* Cs-4 and *C. sinensis* compartments relative to those between the *H. sinensis* Bailing and *C. sinensis* compartments (*cf*. Table 2). The cluster relationship demonstrated by the semi-quantitative neighbor-joining algorithm was validated using the fully quantitative approach of the furthest neighbor algorithm without the bootstrap strategy provided by the SPSS software (*cf.* Figure 8).

Local herbal farmers in the *C. sinensis* production areas of the Qinghai-Tibetan Plateau have long recognized the temperature dependency of the *C. sinensis* maturational features and believe that “eating ‘worms’ in the winter and ‘grass’ in the summer” provides tonic herbal properties. Changes in the therapeutic properties of *C. sinensis* during its maturation have also been recognized by the field of traditional Chinese medicine, in which natural *C. sinensis* is graded accordingly. We have reported maturational changes in the composition of multiple intrinsic fungal species of *C. sinensis* and at least 6 genotypes of *O. sinensis*, along with environmental changes (temperature, sunlight intensity, snow/rain, moisture and plateau wind) on the Qinghai-Tibetan Plateau [13],[19]–[23]. The altered fungal background of natural *C. sinensis* at various maturation stages causes large variations in (i) the RAPD molecular marker polymorphisms, (ii) the fingerprints of small organic compounds, and (iii) proteomic polymorphisms in the caterpillar bodies and stromata, as demonstrated in this and previous studies [13],[19]–[23],[28]–[29]. The integration of the component compounds that are differentially expressed in different compartments of *C. sinensis* and differentially altered during *C. sinensis* maturation constitutes the dynamic pharmacological base that is responsible for the varying potencies of the health benefits and therapeutic activities associated with *C. sinensis*.

In conclusion, SELDI-TOF MS proteomic profiling was used to macrocosmically detect the dynamic polymorphic alterations among differentially expressed proteins in the different *C. sinensis* compartments during maturation. The apparent proteomic polymorphism dissimilarity between *H. sinensis* and *C. sinensis* suggests different fungal backgrounds of these organisms and thus might not support the “single-fungus” hypothesis of *C. sinensis* or the hypothesis of an anamorph-teleomorph connection between *H. sinensis* and natural *C. sinensis*. However, the findings from this proteomic study, in corroboration with prior mycological and molecular observations, support the integrated micro-ecosystem hypothesis for natural *C. sinensis*.
